# CircularRNA_0119872 regulates the microRNA-582-3p/E2F transcription factor 3 pathway to promote the progression of malignant melanoma

**DOI:** 10.6061/clinics/2021/e3036

**Published:** 2021-09-28

**Authors:** Jinlong Qu, Chunying Yuan, Qi Jia, Mengwei Sun, Min Jiang, Fuguang Zuo

**Affiliations:** I Department of Dermatology, Linyi Central Hospital, Linyi 276400, Shandong, China.; II Department of Dermatology, Dongying People's Hospital, Dongying 257091, Shandong, China.; III Department of Dermatology, Haiyang People’s Hospital, Haiyang 265100, Shandong, China.

**Keywords:** Melanoma, Circ_0119872, miR-582-3p, E2F3

## Abstract

**OBJECTIVES:**

Malignant melanoma (MM) is an invasive tumor that poses a threat to patient health. Circular RNAs (circRNAs) are important regulators of MM carcinogenesis. In this study, we investigated the expression characteristics and biological functions of, and mechanism underlying, circ_0119872 expression in MM.

**METHODS:**

Quantitative reverse transcription-polymerase chain reaction (qRT-PCR) was employed to examine the circ_0119872, microRNA (miR)-582-3p, and E2F transcription factor 3 (E2F3) mRNA expression levels in MM tissues and cell lines. Western blotting was performed to quantify E2F3 protein expression. MM cells with circ_0119872 knockdown were established, and cell counting kit 8 (CCK-8) and transwell assays were utilized to examine the function of circ_0119872 and its effects on the malignant characteristics of MM cells. The MiRDB and TargetScan databases were used to predict the target genes of miR-582-3p. Kyoto Encyclopedia of Genes and Genomes (KEGG) pathway analysis was used to explore the biological functions of the target genes of miR-582-3p. Additionally, a dual-luciferase reporter gene experiment was performed to verify the targeting relationship between circ_0119872 and miR-582-3p as well as that between miR-582-3p and E2F3.

**RESULTS:**

Circ_0119872 was remarkably upregulated in MM tissues and cell lines. Circ_0119872 knockdown suppressed the cell proliferation and metastasis In addition, miR-582-3p was identified as a downstream target of circ_0119872. The target genes of miR-193a-3p are involved in melanogenesis and cancer-related signaling pathways. Mechanistically, circ_0119872 facilitated MM progression by adsorbing miR-582-3p and upregulating E2F3 expression.

**CONCLUSION:**

Circ_0119872 is an oncogenic circRNA that participates in the promotion of MM progression by regulating the miR-582-3p/E2F3 axis.

## INTRODUCTION

Malignant melanoma (MM) is a malignancy that originates in melanocytes (1). Statistically, there are more than 200,000 new cases of MM worldwide each year, and its morbidity is increasing annually (2,3). Identifying the mechanisms of MM development is essential for the development of novel drugs to treat this aggressive disease.

Circular RNAs (circRNAs) are non-coding RNAs with covalently bonded closed-loop structures (4-6). Over the past few years, with the development of high-throughput sequencing technology and bioinformatics, circRNAs have attracted widespread attention (5). CircRNAs are promising biomarkers and therapeutic targets for tumors. For example, circ_0000267 is an oncogenic circRNA that regulates the progression of gastric cancer (6). Multiple circRNAs, such as circ_0084043 (4), circ_0016418 (5), and circ_0025039 (7), are aberrantly expressed in MM tissues and participate in tumorigenesis and metastasis. Nonetheless, the functions of most circRNAs in MM remain unclear. Therefore, there is a need to further identify the roles and underlying mechanisms of circRNAs in the development of MM.

MicroRNAs (miRNAs) are implicated in tumorigenesis by binding to the 3′-untranslated region (UTR) of target mRNA. For instance, diverse miRNAs can participate in MM development as tumor-suppressive factors, such as miR-488-5p, which facilitates MM development by regulating the Wnt/β-catenin pathway (8). MiR-137 restrains glutamine catabolism and MM cell proliferation by targeting glutaminase (9). MiR-596 represses MM cell proliferation, migration, and invasion and enhances apoptosis by blocking the mitogen-activated protein kinase (MAPK)/extracellular signal‐regulated kinase (ERK) signaling pathway (10). Previous research has confirmed that miR-582-3p suppresses the progression of diverse tumors, such as prostate cancer (11), gastric cancer (12), and acute myeloid leukemia (13). However, the role of miR-582-3p in MM has not yet been fully elucidated.

E2F transcription factor 3 (E2F3), one of the major members of the E2F family, is crucial in modulating the cell cycle progression, proliferation, and apoptosis (14). High E2F3 expression has been reported to be associated with worse prognosis in patients with MM and is implicated in MM progression as an oncogene (15). In this study, we identified a novel circRNA, circ_0119872, whose expression was remarkably upregulated in MM tissues and cell lines. We confirmed that circ_0119872 adsorbed miR-582-3p to upregulate E2F3 expression to facilitate MM progression, which implies that circ_0119872 might be a potential target for MM therapy.

## MATERIALS AND METHODS

### Subjects and tissue specimens

Thirty cases of MM tumor tissues and paracancerous normal tissues were obtained from Linyi Central Hospital from July 2016 to March 2019, and all specimens were confirmed by pathologists. The specimens were preserved in liquid nitrogen immediately after their removal. This study was approved by the Ethics Committee of Linyi Central Hospital (Approval No. 201601005) and performed in accordance with the Declaration of Helsinki.

### Cell culture

Four MM cell lines (A357, A875, MUM-2B, and M21) and normal human epidermal melanocytes (HEMa-LP cells) were procured from the American Type Culture Collection (ATCC, Bethesda, MD, USA) and the Cell Bank of the Chinese Academy of Sciences (Shanghai, China). All cells were cultured in the Roswell Park Memorial Institute (RPMI)-1640 medium (Sangon, Shanghai, China) containing fetal bovine serum (FBS, 10%; Gibco, NY, USA), penicillin (100 U/mL; Gibco, NY, USA), and streptomycin (100 μg/mL; Gibco, NY, USA) at 37°C in 5% carbon dioxide (CO_2_).

### Cell transfection

Two small interfering RNAs (siRNAs) of circ_0119872 (si-circ_0119872#1: 5′-GAGATGTTTGGTTCTCCAAAA-3′ and si-circ_0119872#2: 5′-GTTTGGTTCTCCAAAATACTT-3′), negative control siRNA (si-NC: 5′- AAGGTGAACTGTCTGGATAAG-3′); and miR-582-3p mimics (5′-UAACUGGUUGAACAACUGAACC-3′), miR-582-3p inhibitors (miR-582-3p in: 5′-GGUUCAGUUGUUCAACCAGUUA-3′), and the corresponding control (miR-NC: 5′-UCACAACCUCCUAGAAAGAGUAGA-3′) were synthesized by Ribobio (Guangzhou, China). When the cells reached 80% confluence, the vectors/oligonucleotides were transfected into the cell lines using Lipofectamine^TM^ 2000 (Invitrogen, Carlsbad, CA, USA) according to the manufacturer’s instructions.

### Quantitative reverse transcription-polymerase chain reaction (qRT-PCR)

Total RNA was isolated from tissues and cell lines using the TRIzol kit (Thermo Fisher Scientific, Inc., Waltham, MA, USA). RNA was reverse transcribed to cDNA using PrimeScript™ RT kit (Takara, Dalian, China). SYBR^®^ Premix Ex TaqTM II Kit (TaKaRa, Dalian, China) and StepOnePlus Real-Time PCR System (Thermo Fisher Scientific, Inc., Waltham, MA, USA) were used to perform qRT-PCR. Finally, the relative expression levels of the genes were calculated using the 2^-ΔΔCt^ method, with glyceraldehyde 3-phosphate dehydrogenase (GAPDH) and U6 as internal references. The primer sequences were as follows: circ_0119872, 5′-TGAGCTTGTGAGTGAGTGGT-3′ (forward) and 5′-GCAAGGAGAATGGCGAGATG-3′ (reverse); miR-582-3p, 5′-GCACACATTGAAGAGGACAGAC-3′ (forward) and 5′-TATTGAAGGGGGTTCTGGTG-3′ (reverse); E2F3, 5′-TATCCCTAAACCCGCTTCC-3′ (forward) and 5′- TTCACAAACGGTCCTTCTA-3′ (reverse); RAS guanyl releasing protein 3 (RASGRP3), 5’-TCTTGGTTTGATTCGTATGA-3′ (forward) and 5′-TTTTTTCCTCTGTGTGACTC-3′ (reverse); U6, 5′-GACTATCATATGCTTACCGT-3′ (forward) and 5′-GGGCAGGAAGAGGGCCTAT-3′ (reverse); GAPDH, 5′-CTTTGGTATCGTGGAAGGACTC-3′ (forward) and 5′-GTAGAGGCAGGGATGATGTTCT-3′ (reverse).

### Subcellular fractionation

RNA was extracted from the nucleus and cytoplasm of MUM-2B and M21 cells, respectively, using the PARIS™ Kit (Thermo Fisher Scientific, Waltham, MA, USA). Then, circ_0119872 enrichment in the nuclear and cytoplasmic RNA was examined by qRT-PCR, with U6 as the nuclear marker and GAPDH as the cytoplasmic marker.

### Cell counting kit-8 (CCK-8) assay

The transfected cells were trypsinized with trypsin, plated in a 96-well plate (2×10^3^ cells/well), and routinely cultured. On the 1^st^, 2^nd^, 3^rd^, and 4^th^ day, 10 μL of CCK-8 reagent (Dojindo Laboratories, Kumamoto, Japan) was added into each well, and the cells were incubated for 2h at 37°C. Next, the absorbance of the cells was measured at 450 nm using a microplate reader (Bio-Rad, Hercules, CA, USA).

### Transwell experiment

Cell migration and invasion experiments were performed using Transwell chambers (pore size: 8 μm) (Thermo Fisher Scientific, Waltham, MA, USA). In cell migration experiment, for each Transwell chamber, 1×10^5^ cells (in 150 µL of serum-free medium) were planted in the top compartment, and 600 μL of complete medium was added to the bottom compartment, and after the incubation for 24h at 37°C, the non-migrated cells in the top compartment were wiped off, and the cells on the bottom surface of the membrane were fixed with 95% ethanol and stained with 0.1% crystal violet for 25 min. Subsequently, the cells were observed and counted under a microscope (Olympus, Tokyo, Japan). Matrigel was used to cover the membranes of the Transwell chambers before the cells were inoculated in the invasion experiment, and the rest of the procedures were the same as in the migration experiment.

### Dual-luciferase reporter gene experiment

The wild-type (WT) and mutant (MUT) sequences of circ_0119872 or E2F3 3′-UTR with potential complementary binding sequences to miR-582-3p were synthesized by Promega (Madison, WI, USA) and cloned into the psiCHECKTM-2-luciferase reporter plasmid (Promega, Madison, WI, USA), and the luciferase reporter plasmids were co-transfected with miR-582-3p mimics or miR-NC into HEK-293T cells. Then, 48h later, the relative luciferase activity of the cells was determined using the dual-luciferase reporter assay system (Promega, Madison, WI, USA) according to the manufacturer’s instructions.

### Western blotting

Total proteins were isolated from cells using the radioimmunoprecipitation assay (RIPA) lysis buffer (Beyotime, Shanghai, China) and quantified using a BCA protein detection kit (Beyotime, Shanghai, China). Then, 30 μg of protein sample from each group was separated by sodium dodecyl sulfate polyacrylamide gel electrophoresis (SDS-PAGE) and then transferred to polyvinylidene fluoride (PVDF) membranes (Millipore, Billerica, MA, USA). Next, the PVDF membranes were blocked with 5% skimmed milk and then incubated with anti-E2F3 antibody (1:1000, ab152126, Abcam Inc., Cambridge, UK) and anti-GAPDH antibody (1:1000, ab9485, Abcam Inc., Cambridge, UK) overnight at 4°C, followed by incubation with horseradish peroxidase-conjugated secondary antibody (1:2000, ab150077, Abcam Inc., Cambridge, UK) for 1h at room temperature. Next, the protein bands were developed using an ECL chemiluminescence substrate kit (Thermo Fisher Scientific, Waltham, MA, USA).

### Bioinformatics analysis

The targeting relationship between circ_0119872 and miR-582-3p was predicted using the CircInteractome database (https://circinteractome.irp.nia.nih.gov/). The target genes of miR-582-3p were predicted using miRDB (http://mirdb.org/) and TargetScan (http://www.targetscan.org/vert_72/) databases. Kyoto Encyclopedia of Genes and Genomes (KEGG) pathway enrichment analyses of the target genes were performed using the web-based tool DAVID v.6.8 (https://david.ncifcrf.gov). The results were visualized using the ggplot2 module in the R software.

### Statistical analysis

The data were collected after all assays were executed three times, and the results are shown as the mean±standard deviation. Statistical analysis was performed using SPSS software v.17.0 (SPSS Inc., Chicago, IL, USA), and the figures were plotted using GraphPad Prism v.5.0 (GraphPad Software, Inc., La Jolla, CA, USA). Shapiro-Wilk normality test and F test were used to verify the normality and equal variance of the data, and the results showed that the data conformed to the normal distribution and homogeneity of variance. Student’s *t*-test was applied for the analysis of differences between two groups, and one-way analysis of variance (ANOVA) was used to analyze differences among three or more groups. Correlations were evaluated using the Pearson’s correlation coefficient. *p<0.05, **p<0.01, and ***p<0.001 were considered to indicate statistical significance.

## RESULTS

### Circ_0119872 expression is remarkably upregulated in MM tissues and cell lines

**Figure 1:**
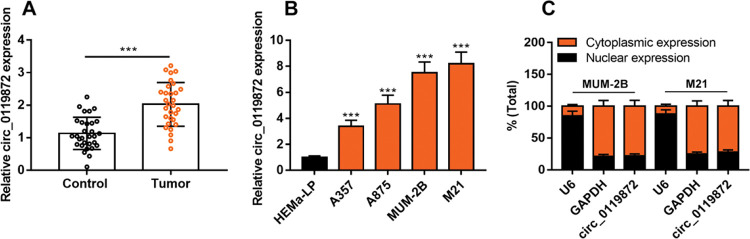
Circ_0119872 expression is remarkably upregulated in malignant melanoma (MM) tissues and cell lines. **(A)** Circ_0119872 expression in 30 pairs of MM and normal skin tissues was examined by quantitative reverse transcription-polymerase chain reaction (qRT-PCR). **(B)** qRT-PCR was performed to detect circ_0119872 expression in normal human epidermal melanocytes (HEMa-LP) and MM cell lines (A357, A875, MUM-2B, and M21). **(C)** Circ_0119872 expression in the nucleus and cytoplasm of MUM-2B and M21 cells was detected by qRT-PCR. U6 and glyceraldehyde 3-phosphate dehydrogenase (GAPDH) served as the controls for the nuclear and cytoplasmic fractions, respectively. ***p<0.001.

Circ_0119872 expression in 30 pairs of MM tissues and normal skin tissues was examined using qRT-PCR, and the data revealed that the mean circ_0119872 expression in MM tissues was 2.2-fold higher than that in normal skin tissues (Figure 1A). Additionally, circ_0119872 was expressed at remarkably higher levels in MM cell lines than in HEMa-LP cells (Figure 1B). Given that circ_0119872 was expressed at the highest level in MUM-2B and M21 cells, these two cell lines were selected for subsequent experiments. The cytoplasm and nucleus of MM cells were isolated to determine the subcellular distribution of circ_0119872. qRT-PCR revealed that circ_0119872 was mainly distributed in the cytoplasm of MUM-2B and M21 cells (Figure 1C). Hence, circ_0119872 might function as a competitive endogenous RNA (ceRNA) to decoy miRNAs and regulate gene expression during MM progression.

### Knockdown of circ_0119872 impedes the proliferation, migration, and invasion of MM cells

**Figure 2:**
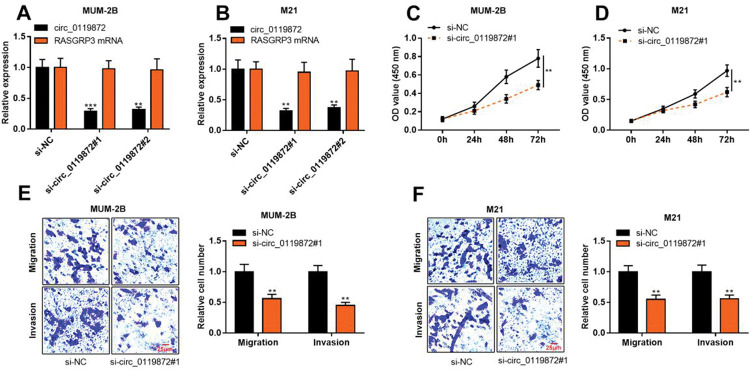
Knockdown of circ_0119872 remarkably represses the proliferation, migration, and invasion of MM cells. **(A,B)** qRT-PCR was used to detect the circ_0119872 and RAS guanyl releasing protein 3 (RASGRP3) mRNA expression levels in MUM-2B and M21 cells after transfection with circ_0119872 small interfering RNA (siRNA). **(C,D)** The cell counting kit 8 (CCK-8) assay was performed to detect the proliferation of MUM-2B and M21 cells after transfection with circ_0119872 siRNA. **(E,F)** Transwell experiments were performed to detect the migration and invasion of MUM-2B and M21 cells after transfection with circ_0119872 siRNA. **p<0.01 and ***p<0.001.

To probe into the regulatory effects of circ_0119872 on the biological behaviors of MM cells, two circ_0119872 siRNAs (si-circ_0119872#1 and si-circ_0119872#2) were transfected into MUM-2B and M21 cell lines, respectively, and the circ_0119872 under-expression models were successfully constructed. Circ_0119872 is produced from the transcript of RASGRP3. As shown, after transfection, circ_0119872 expression in MM cells was reduced by approximately 70%, while RASGRP3 mRNA expression was not markedly affected (Figure 2A, B). si-circ_0119872#1 was selected for subsequent experiments. CCK-8 assay was performed to detect cell proliferation, and the data revealed that the proliferation of MUM-2B and M21 cells was significantly inhibited in si-circ_0119872#1 group, compared with the control group (Figure 2C, D). The data of the Transwell experiment revealed that the transfection of si-circ_0119872#1 inhibited the migration and invasion of MM cells by nearly 50% (Figure 2E, F). The above data implied that circ_0119872 could regulate the malignant phenotypes of MM cells.

### Circ_0119872 positively regulates E2F3 expression by repressing miR-582-3p

**Figure 3:**
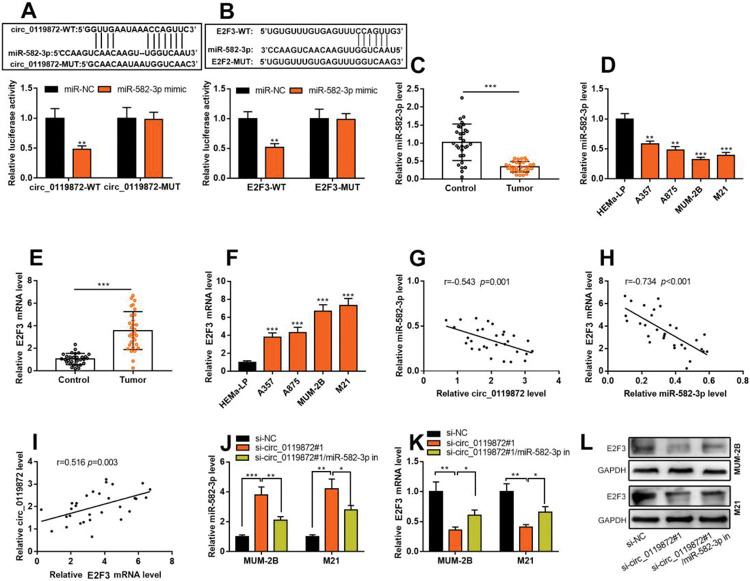
Circ_0119872 modulates the expression of E2F transcription factor 3 (E2F3) through miR-582-3p. **(A,B)** Bioinformatics analysis and dual-luciferase reporter assay confirmed the direct binding relationship between circ_0119872 and miR-582-3p as well as that between miR-582-3p and E2F3 mRNA 3′-untranslated region (UTR). **(C)** MiR-582-3p expression in 30 pairs of MM and normal skin tissues was examined by qRT-PCR. **(D)** qRT-PCR was conducted to examine miR-582-3p expression in normal human epidermal melanocytes and MM cell lines (A357, A875, MUM-2B, and M21). **(E)** E2F3 expression in 30 pairs of MM and normal skin tissues was examined by qRT-PCR. **(F)** qRT-PCR was performed to examine E2F3 expression in normal human epidermal melanocytes and melanoma cell lines (A357, A875, MUM-2B, and M21). **(G-I)** Pearson correlation analysis was performed to analyze the correlation between circ_0119872, miR-582-3p, and E2F3 expression levels in MM tissues. **(J)** MiR-582-3p expression in MUM-2B and M21 cell lines was detected by qRT-PCR after co-transfection with si-circ_0119872 #1 and miR-582-3p inhibitors. **(K,L)** qRT-PCR and western blotting were conducted to detect E2F3 expression in MUM-2B and M21 cell lines after co-transfection with si-circ_0119872#1 and miR-582-3p inhibitors. *p<0.05, **p<0.01, and ***p<0.001.

**Figure S1:**
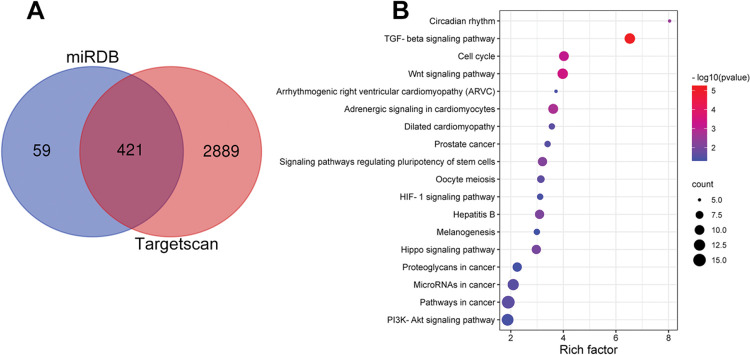
The prediction and functional annotation of target genes for microRNA (miR)-582-3p. (**A**) Venn diagrams showing the intersection between the predicted target genes of miR-582-3p from the two databases. (**B**) Kyoto Encyclopedia of Genes and Genomes (KEGG) pathway enrichment analysis of the target genes of miR-582-3p.

Notably, bioinformatics analysis predicted that there were complementary binding sites between the circ_0119872 sequence and miR-582-3p sequence, and between miR-582-3p sequence and E2F3 3′-UTR sequence. To validate the binding relationships between circ_0119872, miR-582-3p, and E2F3 3′-UTR, dual-luciferase reporter gene experiments were performed, and the data revealed that the luciferase activity of HEK-293T cells in the circ_0119872-WT+miR-582-3p mimic group was reduced by nearly 50% compared to that in the circ_0119872-WT+miR-NC group, but miR-582-3p overexpression did not significantly reduce the luciferase activity of the MUT reporters (Figure 3A). Similarly, the luciferase activity of HEK-293T cells in the E2F3-WT+miR-582-3p mimic group was reduced by nearly 50% compared to that in the E2F3-WT+miR-NC group, but miR-582-3p overexpression did not significantly reduce the luciferase activity of the MUT reporters (Figure 3B). In addition to E2F3, the miRDB database predicted a total of 480 target genes of miR-582-3p, and TargetScan predicted 3310; 421 target genes were obtained from the intersection of the two sets (Figure S1A). To further analyze the biological functions of the circ_0119872/miR-582-3p axis, the potential biological functions of these target genes were predicted using KEGG pathway analyses. The results of KEGG analyses showed that these target genes were mainly associated with melanogenesis and cancer-related pathways (Figure S1B), which indicated that circ_0119872/miR-582-3p could promote the progression of MM by regulating the expression of downstream genes. Moreover, qRT-PCR revealed that miR-582-3p expression was remarkably downregulated in MM tissues and cell lines (Figure 3C, D). In addition, E2F3 mRNA expression was markedly upregulated in MM tissues and cell lines (Figure 3E, F). Pearson correlation analysis indicated that miR-582-3p expression in MM tissues was negatively correlated with circ_0119872 expression and negatively correlated with E2F3 mRNA expression, while circ_0119872 and E2F3 mRNA expression were positively correlated (Figure 3G-I). qRT-PCR and western blotting showed that miR-582-3p expression in MM cells of si-circ_0119872#1 group was augmented by approximately 4-fold compared with that in the si-NC group, and E2F3 mRNA and protein expression in si-circ_0119872#1 group was reduced by at least 60% of that in the si-NC group. Notably, inhibiting miR-582-3p partially counteracted the regulatory effects of circ_0119872 knockdown on the expression of miR-582-3p and E2F3 (Figure 3J-L). Collectively, the above findings revealed that circ_0119872 modulated E2F3 expression through miR-582-3p in MM cells.

### Inhibition of miR-582-3p reverses the inhibitory effects of circ_0119872 knockdown on the malignant biological behaviors of MM cells

**Figure 4:**
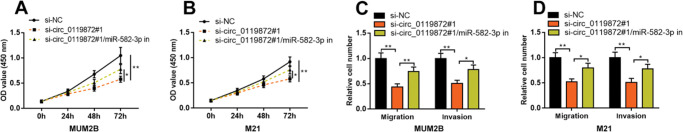
MiR-582-3p inhibitors partially reverse the inhibitory effects of the knockdown of circ_0119872 on MM cell proliferation, migration, and invasion. **(A,B)** CCK-8 assay was employed to detect the MM cell proliferation after co-transfection with si-circ_0119872#1 and miR-582-3p inhibitors. **(C,D)** Transwell assay was performed to detect the MM cell migration and invasion after co-transfection with si-circ_0119872#1 and miR-582-3p inhibitors. *p<0.05 and **p<0.01.

To confirm that circ_0119872 exerts its biological function in MM progression by repressing miR-582-3p, “rescue experiments” were performed. CCK-8 and Transwell assays indicated that circ_0119872 knockdown suppressed the proliferation, migration, and invasion of MUM-2B and M21 cells, and the co-transfection of miR-582-3p inhibitors partially counteracted these suppressive effects (Figure 4A-D). The above data substantiated that circ_0119872 exerted an oncogenic effect on MM progression by modulating miR-582-3p.

## DISCUSSION

Accumulating evidence confirms that circRNAs are closely linked to MM development. For example, circ_0084043, whose expression is remarkably upregulated in MM tissues, enhances MM cell proliferation, migration, and invasion by regulating the miR-153-3p/Snail axis (4); circ_0016418, whose expression is remarkably upregulated in MM tissues and cell lines, facilitates MM cell proliferation, migration, and epithelial-mesenchymal transition (EMT) by suppressing miR-625 expression and upregulating YY1 expression (5); and circ_0025039 facilitates MM cell proliferation, invasion, and glucose metabolism by modulating the miR-198/CDK4 axis (16). Circ_0119872 is a circRNA derived from the transcript of the RASGRP3 gene, located on chromosome chr2:33736776-33740273. A recent study reported that circ_0119872 promotes the malignancy of uveal melanoma cells by regulating the miR-622/G3BP1 axis (17). In the present study, circ_0119872 was found to be expressed at remarkably higher levels in MM tissue specimens than in normal tissues, and knocking down circ_0119872 suppressed the proliferation, migration, and invasion of MM cells, which is consistent with a previous report (17).

MiR-582-3p, a tumor-suppressive miRNA, inhibits the progression of diverse tumors. For instance, miR-582-3p expression is remarkably downregulated in bone metastatic prostate cancer tissues, and miR-582-3p under-expression is linked to poor prognosis of the patients; miR-582-3p targets the TGF-β signaling pathway to impede cell migration, invasion, and bone metastasis of cancer cells (11). MiR-582-3p impedes gastric cancer cell proliferation, migration, and invasion by suppressing HUR expression (12). In addition, miR-582-3p expression is remarkably downregulated in acute myeloid leukemia and miR-582-3p represses cell proliferation and induces G2/M cell arrest by targeting cyclin B2 (13). In this study, circ_0119872 was found to be mainly distributed in the cytoplasm of MM cells, so it was assumed that circ_0119872 might function as a ceRNA. As mentioned above, miR-622 has been identified as a target of circ_0119872 in MM cells (17). In the present study, bioinformatics and dual-luciferase reporter gene experiments verified that miR-582-3p also specifically bound to circ_0119872. Additionally, miR-582-3p was found to be underexpressed in MM tissues and cell lines. In addition, the miR-582-3p inhibitor partially counteracted the suppressive effects of circ_0119872 knockdown on MM cell proliferation, migration, and invasion. These results suggest that miR-582-3p is a novel target of circ_0119872.

The E2F3 gene is located on chromosome 6, NC_000006.12, as a member of the E2F family with two distinct isoforms, E2F3a and E2F3b (18,19). E2F3a is a transcriptional activator that is essential for cell proliferation, and E2F3b is a repressor that impedes the transcription of the target genes of E2F. E2F3, E2F3a, and E2F3b exert synergistic or antagonistic roles in tumorigenesis and progression (18,19). Dysregulation of E2F3 expression is linked to diverse diseases, such as age-related cataracts (20) and Hirschsprung’s disease (21). Additionally, E2F3 has been implicated in the development of diverse tumors as oncogenes. For instance, in endometrial cancer, HOXB9 enhances cancer cell migration by upregulating the expression of E2F3 (22). In cervical cancer, TTN-AS1 enhances cancer cell proliferation, migration, and invasion by repressing miR-573 and upregulating E2F3 (23). In bladder cancer, depletion of RB transcriptional corepressor 1 activates the E2F3, Myc, and mTOR signaling pathways to facilitate proliferation and restrain apoptosis of cancer cells (24). Reportedly, in MM, the copy number of the E2F3 gene is amplified, and the copy number is positively correlated with its expression, and patients with a high copy number have a poor prognosis, and E2F3 enhances MM cell proliferation and facilitates MM progression (25). Moreover, E2F3 enhances the migration and invasion of MM cells (26). Our work confirmed that E2F3 expression was remarkably upregulated in MM tissues and cell lines. We also demonstrated that the circ_0119872/miR-582-3p axis contributes to the dysregulation of E2F3 expression in MM.

**Figure 5:**
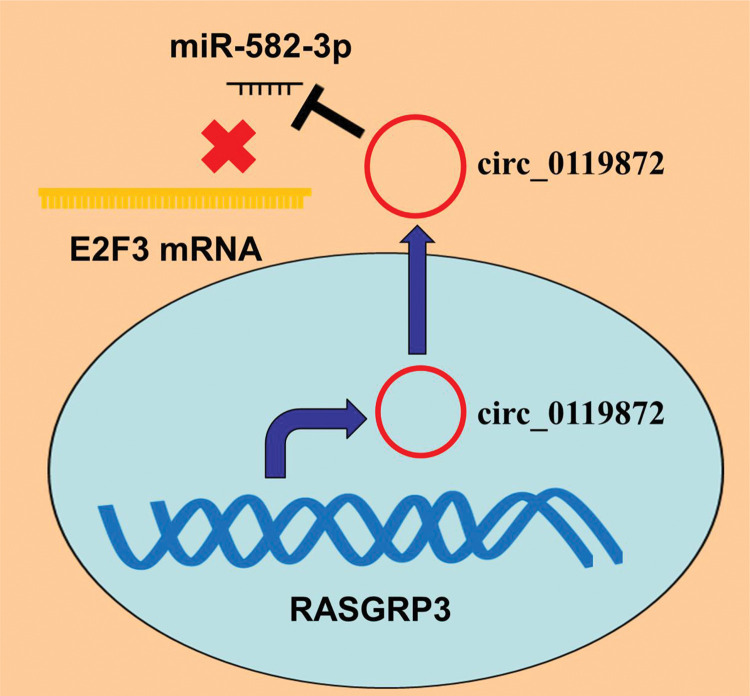
Graphic Abstract: circ_0119872 enhances MM progression by adsorbing miR-582-3p and upregulating the expression of E2F3.

In conclusion, this study confirmed that the ceRNA network consisting of circ_0119872, miR-582-3p, and E2F3 is involved in MM progression (Figure 5). Therefore, our study presents a new mechanism to explain the progression of MM and provides novel clues for the diagnosis and treatment of MM.

## AUTHOR CONTRIBUTIONS

Qu J and Zuo F were responsible for the study conception and design. Qu J, Yuan C, Jia Q and Zuo F were responsible for the development of methodology. Sun M and Jiang M were responsible for the acquisition of data. Qu J, Yuan C and Jia Q were responsible for the analysis and interpretation of data. Qu J and Zuo F were responsible for manuscript writing and revision.
